# Supplementary material for: Identification of photoperiod-induced specific miRNAs in the adrenal glands of Sunite sheep (Ovis aries)
Source: Front Vet Sci. 2022 Jul 22;9:888207. doi: 10.3389/fvets.2022.888207 (PMC9354845; doi:10.3389/fvets.2022.888207)
Supplement: Supplementary file 1 [file Data_Sheet_1.ZIP › Supplementary file/miRDeep2 file.zip!/miRDeep2 file/Know_Structure/SP42A2/oar-mir-30d.pdf]

miRBase precursor : oar-mir-30d
Total read count : 209909
oar-miR-30d read count : 209227
remaining reads : 682

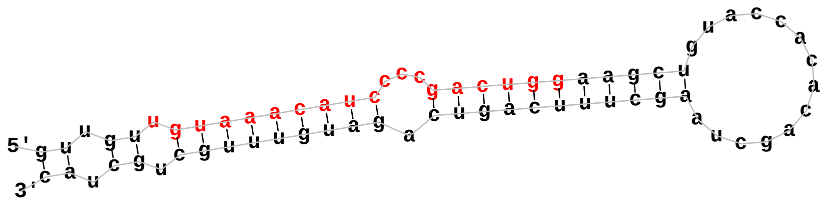

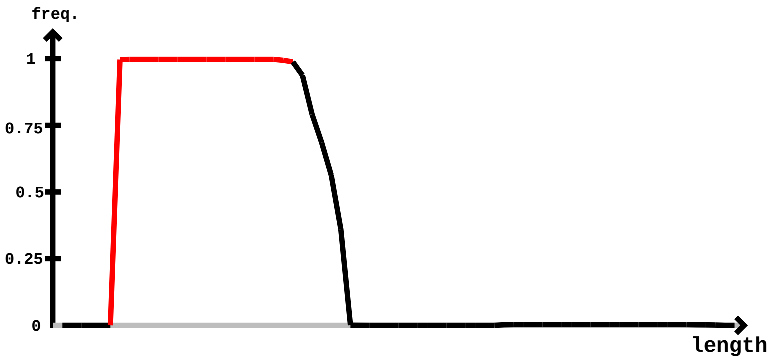


```
       oar-miR-30d
5'- guuguuguaaacauccccgacuggaagcuguaccacacagcuaagcuuucagucagauguuugcugcuac   -3'    exp
    ...((.(((((((((...(((((((((((..............))))))))))).))))))))).))...    reads   mm   sample
    ....uuguaaacauccccgacugga.............................................    1       0    seq
    ....uuguaaacauccccgacuggaa............................................    1       0    seq
    ....uuguaaacauccccgacuggaagcu.........................................    1       0    seq
    .....uguaaacauccccgacu................................................    741     0    seq
    .....uguaaacaAccccgacu................................................    1       1    seq
    .....uguaaacaucUccgacu................................................    5       1    seq
    .....uguaaacaucccUgacu................................................    12      1    seq
    .....uguaaacauccccgGcu................................................    4       1    seq
    .....uguaaacauccccgacug...............................................    1218    0    seq
    .....uguaaacauccccgacugg..............................................    10565   0    seq
    .....uguaaacauccccgacugga.............................................    30614   0    seq
    .....uguaaacauccccgacuggaa............................................    22301   0    seq
    .....uguaaacauccccgacuggaag...........................................    25624   0    seq
    .....uguaaacauccccgacuggaagc..........................................    42599   0    seq
    .....uguaaacauccccgacuggaagcu.........................................    75404   0    seq
    .....uguaaacauccccgacuggaagcug........................................    67      0    seq
    .....uguaaacauccccgacuggaagcugu.......................................    42      0    seq
    .....uguaaacauccccgacuggaagcugua......................................    1       0    seq
    .....uguaaacauccccgacuggaagcuguac.....................................    3       0    seq
    .....uguaaacauccccgacuggaagcuguacca...................................    1       0    seq
    ......guaaacauccccgacugg..............................................    3       0    seq
    ......guaaacauccccgacugga.............................................    15      0    seq
    ......guaaacauccccgacuggaa............................................    13      0    seq
    ......guaaacauccccgacuggaag...........................................    14      0    seq
    ......guaaacauccccgacuggaagc..........................................    25      0    seq
    ......guaaacauccccgacuggaagcu.........................................    58      0    seq
    .......uaaacauccccgacuggaa............................................    1       0    seq
    .......uaaacauccccgacuggaagc..........................................    2       0    seq
    .......uaaacauccccgacuggaagcu.........................................    5       0    seq
    ...........................................agcuuucagucGgauguuu........    1       1    seq
    .............................................cuuucagucagauguuugc......    21      0    seq
    .............................................cuuucagucagauguuugcu.....    103     0    seq
    .............................................cuuucagucagauguuugcug....    52      0    seq
    .............................................cuuucagucagauguuugcugc...    98      0    seq
    .............................................cuuucagucagauguuugcugcu..    146     0    seq
```

oar-miR-30d

```
guuguuguaaacauccccgacuggaagcuguaccacacagcuaagcuuucagucagauguuugcugcuac
.............................................cuuucagucagauguuugcugcua.    1    0    seq
..............................................uuCcagucagauguuugcu.....    1    1    seq
..............................................uuucagucagauguuugcu.....    18   0    seq
..............................................uuucagucagauguuugcug....    15   0    seq
..............................................uuucagucagauguuugcugc...    35   0    seq
..............................................uuucagucagauguuugcugcu..    74   0    seq
..............................................uuucagucagauguuugcugcua.    1    0    seq
...............................................uucagucagauguuugcugc...    1    0    seq
................................................ucagucagauguuugcugc...    1    0    seq
```
